# Supplementary material for: Thirteen dubious ways to detect conserved structural RNAs
Source: IUBMB Life. Author manuscript; Available in PMC 2024 Jul 10. (PMC11234323; doi:10.1002/iub.2694)
Supplement: supplementary_material [file NIHMS1907110-supplement-supplementary_material.gz › supplemental_material/Table1/CRS.structural.tar.gz!/selected/selected_noRfam/noNUMTs/cmf.M2299369/MyM2299369/R-scape_nhmmer/cmf.M2299369_ext_liftover.hg38.R2R.sto.pdf]

cmf.M2299369_ext_liftover.hg38

5′-○ ○ ○ U C U G G ○ ○ ○ ○ ○ ● C C ● G G G U G ● A ● R ● R R A G G R G G Y G G ● R Y Y C A G C ● C R G A G G G G C U G G A Y U U G G A C U ● ● G R C ● ● C A G G U A C U C R G A G C A A A U G G U G R G A Y C A G A A G G R G G A U U A U G ● C A Y U C C U U C G A A G G A A U G A A U U A A A C G U G C Y U
